# Does narrative drive dynamic attention to a prolonged stimulus?

**DOI:** 10.1186/s41235-018-0140-5

**Published:** 2018-12-07

**Authors:** Stephen J. Hinde, Tim J. Smith, Iain D. Gilchrist

**Affiliations:** 1 School of Psychological Science, University of Bristol, Bristol, UK; 2 Birkbeck, University of London, London, UK

**Keywords:** Attention, Sustained attention, Prolonged attention, Dual-task, Film, Dynamic attention, Temporal attention, Attentional load

## Abstract

Attention in the “real world” fluctuates over time, but these fluctuations are hard to examine using a timed trial-based experimental paradigm. Here we use film to study attention. To achieve short-term engagement, filmmakers make use of low-level cinematic techniques such as color, movement and sound design to influence attention. To engage audiences over prolonged periods of time, narrative structure is used. In this experiment, participants performed a secondary auditory choice reaction time (RT) task to measure attention while watching a film. In order to explore the role of narrative on attention, we manipulated the order that film segments were presented. The influence of narrative was then compared to the contribution of low-level features (extracted using a computer-based saliency model) in a multiple regression analysis predicting choice RT. The regression model successfully predicted 28% of the variance in choice RT: 13% was due to low-level saliency, and 8% due to the narrative. This study shows the importance of narrative in determining attention and the value of studying attention with a prolonged stimulus such as film.

## Significance statement

People in the USA may spend over half their leisure time watching film and TV (Chamorro-Premuzic, Kallias, & Hsu, 2013), with worldwide cinema box office revenues at US$30 billion. Add to this that a full 80% of Americans also spend large amounts of time watching media over the Internet (Chamorro-Premuzic et al., 2013) and this shows that attention paid to visual media is of wide public interest. Audiences appear to find media a compelling experience that effortlessly holds their attention for long periods of time; therefore, studying how attention works by watching how people watch media is an important research topic. The study explored the extent to which attention in cinema is governed by narrative and the audio-visual features of the film, using a dual-task methodology to measure attention.

“The stream of our thought is like a river. On the whole, easy simple flowing predominates in it, the drift of things is with the pull of gravity, and effortless attention is the rule. But at intervals an obstruction, a set-back, a log-jam occurs, stops the current, creates an eddy, and makes things temporarily move the other way.” James (1890, p. 451).

Attention has been a central topic of psychology since before the birth of the modern discipline (James, 1890). Despite the huge progress that has been made in understanding this fundamental cognitive capacity (Allport, 1993; Posner, 2016) many questions remain unanswered and under researched. One constraint that has determined the focus of effort by attention researchers has been the ubiquitous use of the experimental trial. In almost all branches of experimental psychology, including studies of attention, behavior is tested in short, apparently uncorrelated, snapshots. The origins of the trial as a fundamental plank of the field’s methodology is two-fold. First, it is a result of the apparatus used: the tachistoscope and then later on the personal computer. In both cases the next trial had to be prepared and this necessitated a pause in the experiment, the so called inter-trial interval. Second, as behavior and response times are inherently noisy (Carpenter, 1991), experiments repeated the same stimuli, allowing for averaging to occur, to get a more stable estimate of the participants’ performance. Such an approach has, however, shifted the focus of research away from the continuous nature of thought, and attention, as described by James (1890) in the quote above.

Outside the laboratory, cognition, and, more specifically, attentional demands, are of course far less quantized and our visual world is more continuous and dynamic. In this paper we set out to study dynamic attentional processes in these more continuous circumstances. One real-life circumstance in which attention appears to be sustained over an extended time period, often with little obvious mind-wandering, is when people watch a movie. Cutting, DeLong, and Nothelfer (2010) and Smith (2012) have argued that film has been constructed to facilitate very prolonged periods of attention. Film then provides one context to study the attentional engagement over longer time periods. Using film stimuli in psychological studies, and, in particular, for studying attention was anticipated by Münsterberg (1916), a tradition picked up by more recent works (e.g., Hinde, Smith, & Gilchrist, 2017; Hochberg & Brooks, 1996; Simons & Levin, 1997; Smith, 2006; Smith & Henderson, 2008; Smith, 2012; Troscianko, Meese, & Hinde, 2012). Film provides a rich, visual and auditory, dynamic environment for the viewer in which typically there is a continuous story or narrative; is repeatable across participants and is also interesting and compelling for participants. This is more akin to the dynamism of a natural scene than more traditional trial-based experiments. However, despite film being a compelling stimulus for attention research that includes many features of more naturalist “real-world” viewing conditions, film is artificially constructed and includes sudden changes in camera angle, zoom, edits, shots and scenes (Smith, 2012). As a result the patterns of results found with film should not be assumed to be identical to the expected behavior in a real-world setting but rather to provide a controlled (if somewhat artificial) model environment in which to study fluctuations in attention over prolonged durations and the role of factors that may influence those fluctuations.

The way that people pay attention to film can be thought of as being determined by both low-level features and high-level properties of the film. Low-level features are the surface qualities of the moving image, these have been shown to be good predictors of the allocation of overt attention (i.e., gaze) in static and dynamic scenes, these features include luminance, chromaticity, edges, contrast and visual clutter (Itti & Koch, 2000; Reinagel & Zador, 1999; Parkhurst, Law, & Niebur, 2002; Tatler, Baddeley, & Gilchrist, 2005; Nuthmann, 2017). In contrast, high-level properties include the narrative within the film and the event structure (Zacks & Tversky, 2001). For movies, the audio sound track also fluctuates in intensity and so has variable (auditory) salience. This may also play an important part in determining fluctuations in attention via both its low-level properties (e.g., loudness) as well as higher-level properties (e.g., the semantics of what is being said; for examples see Boltz, 2004; Batten & Smith, 2018).

**Fig. 1:**
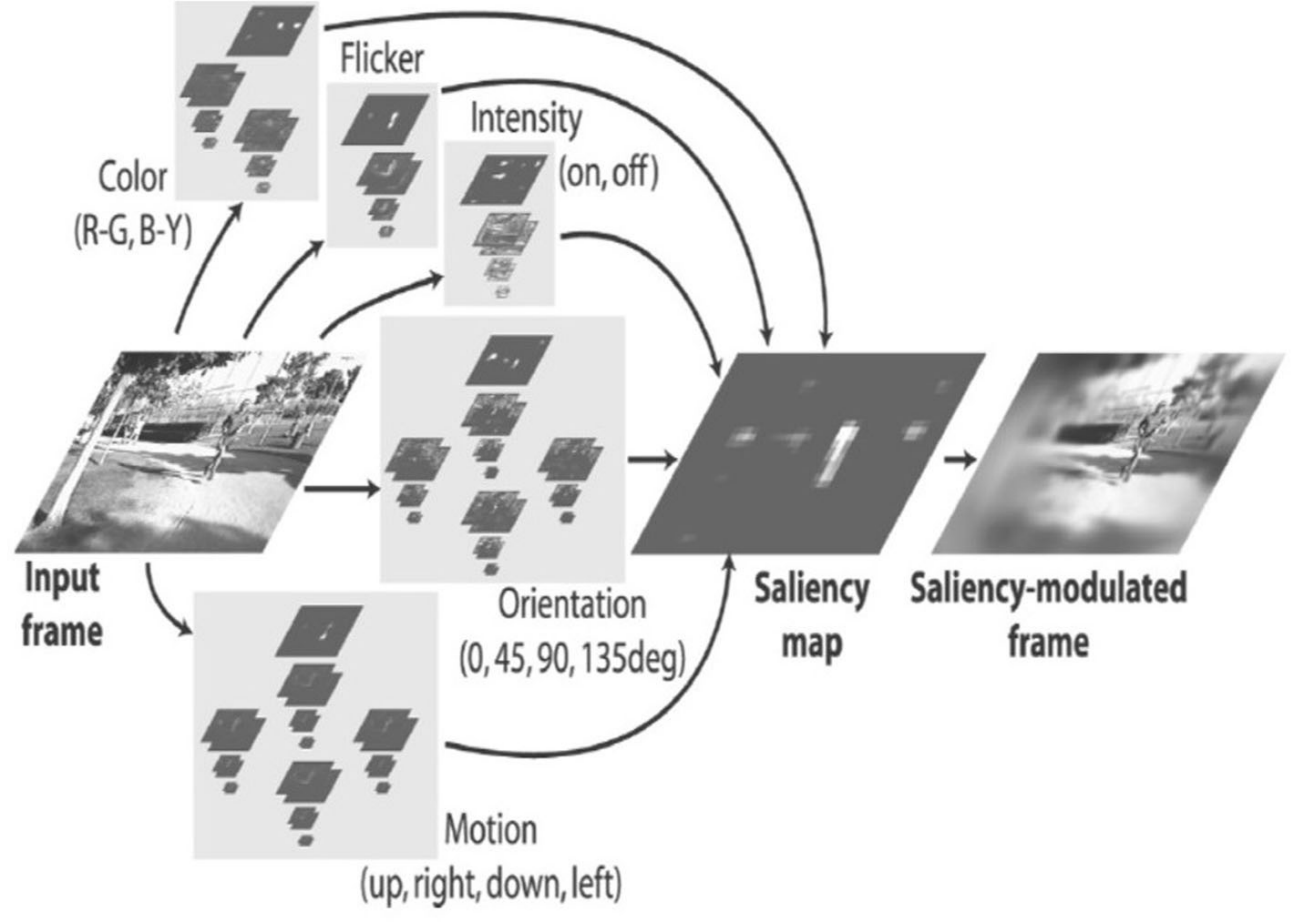
iNVT (2014) biologically inspired computational model (Itti & Koch, 2000)

We will first turn to the evidence that low-level features influence where attention is focused on a scene. The specific hypothesis is that areas on the scene that have higher levels of intensity (or salience) based on a set of low-level features will attract attention. In a series of papers, Itti and others (e.g., Itti, Koch, & Niebur, 1998; Itti & Koch, 2000) have developed a biologically inspired saliency model that can predict the spatial allocation of attention in a scene. Many different saliency models now exist. In a review and meta-analysis, Itti and Borji (2014) concluded that most of these different models make good, but slightly different predictions. One current iteration of the model developed by Itti et al. is the iLab Neuromorphic Vision Toolkit (Itti et al., 2014), henceforth referred to as iNVT, which we will use in the current paper. The architecture of iNVT (Itti et al., 2014) is shown in Fig. 1. There are 72 basic spatial and time-dependent features simulating low-level human vision. These features form a set of center-surround detectors, covering the saliency of different spatial and temporal features at different degrees of scale, direction of motion and temporal dynamics. The feature set includes color detectors, for blue-yellow and red-green color opponency, contrast detectors, intensity, orientation, motion and flicker. In order to achieve different degrees of scale, iNVT (Itti et al., 2014) uses a low-pass spatial filter, applied progressively, to generate image reduction from 1:1 pixels to 1:256 pixels in eight steps, creating a Gaussian dyadic pyramid, containing fine to coarse details of the scene. The pyramid is then processed for the distinct features, using Gabor filters and center-surround feature detectors, applied across fine to coarse maps. This mirrors the functions of the neurons in the lateral geniculate nucleus (LGN), and visual cortex which act as low-level visual-feature detectors. In summary, the biologically inspired algorithm within the model, is designed to mirror the human visual system, generating a unique set of 72 distinct features for the visual scenes. As a result the model, and, in particular, the output of these maps, provides a method to compute the visual salience of a video sequence for different visual features at different spatial scales.

High-level properties also play a key role in the allocation of attention. In the now classic demonstration, Yarbus (1967) showed that viewing behavior on the same static image was radically different under different task instructions. In a similar manner, for moving environments, drivers and passengers look at different parts of the same scene dependent on the task they are allocated (Wallis & Bülthoff, 2000) and Land et al. have demonstrated, using a number of everyday tasks, that attentional allocation is driven by relevance rather than salience (see Land & Furneaux, 1997). In a walking task conducted in Virtual Reality (VR), Jovancevic, Sullivan, and Hayhoe (2006) showed that attention was not drawn to an unexpected pedestrian that suddenly appeared on a collision course with the participant, suggesting that salience played little or no role in this real-world task. This trade-off between low-level and high-level determinates for attention is widely reported (Henderson & Hollingworth, 1999; Hernandez-Peon, Scherrer, & Jouvet, 1956; James, 1890).

When participants are engaged in a more natural continuous task the allocation of attention is determined by the relative balance between high-level properties and lower-level features of the stimulus. For example, the viewing task can influence gaze behavior while watching videos (Smith & Mital, 2013); in natural walking tasks, meaning and reward can be more important than visual saliency (Cristino & Baddeley, 2009) and for dynamic naturalistic scenes, that low-level feature predictors of attention can be overridden by the high-level features (Carmi & Itti, 2006). Wischnewski, Belardinelli, Schneider, and Steil (2010) developed a human-inspired theory of attention for robotic applications which integrated three levels of influence: low-level, static-driven saliency, dynamic visual factors and task-driven factors. One default task that may drive attention over prolonged periods is the perception and comprehension of naturalistic events such as watching a person wash a car, fold laundry or, in the case of more dramatic cinematic narratives, chase and catch a “baddie” (Zacks & Tversky, 2001).

The Event Theory of Zacks and Tversky (2001) provides a framework to understand the influence of high-level properties on attention. Within this theory the continuous stream of dynamic information presented to the senses can be thought of as being structured by related units of information, known as events. Events have construct validity, in that different observers can agree on where the event boundaries should be placed. These boundaries sometimes relate to the task, sometimes to a linguistic boundary, but sometimes seem arbitrary. Research into event structure (Brewer & Dupree, 1983; Zacks, Speer, Swallow, Braver, & Reynolds, 2007) proposes that event structure is important in determining resource allocation mechanisms for perception, attention and memory. For instance, Brewer and Dupree (1983) studied memory retrieval of actions in video and showed that actions within schema were more easily recalled than those that were not.

Measuring the influence of prolonged, high-level factors, such as events on attention, requires methods beyond the traditional trial-based stimulus/response methods used in studies of attention. For example, Hasson, Nir, Levy, Fuhrmann, and Malach (2004) used inter-subject correlations of BOLD signals to study attention in movies and Smith and Henderson (2008) used eye tracking to study selective attention to movies. Another experimental method that has proved to be very fruitful in the study of prolonged attention is the dual-task paradigm (Bezdek & Gerrig, 2016; Kahneman, 1973; Lang, 2000). The dual-task paradigm requires participants to carry out two tasks concurrently, usually labeled the primary and the secondary tasks, fluctuations in performance in the secondary task are taken as evidence that attentional resources are being focused on the primary task to the detriment of the secondary task. The underlying assumption here is that there is a limited capacity of attention, held in a central general pool. As a consequence the more demanding the primary task, the worse the performance on the secondary task due to a deficit in remaining resources. This paradigm was used in early pioneering experiments in cognitive psychology to study a wide range of tasks such as mental arithmetic, reading, and human-computer interface design (Posner & Boies, 1971). The dual-task method remains a simple method to study attention, and has found some renewed interest for use with studying attention and film. For instance, Lang (2000) used dual-task to study attention in short media messages used in television advertisements. Bezdek and Gerrig (2016) used the dual-task paradigm to study the narrowing of attention, during moments of high suspense in film clips. They also looked at how this attentional narrowing, corresponded to transport, defined as shifting attention from the everyday world to the narrative world of the story (Green & Brock, 2002).

In this study we used the dual-task paradigm to study attention while participants viewed extended and continuous segments of a movie. The dual-task allowed us to probe the changes in attentional engagement over time during the movie. There were two main research questions addressed by the paper. Firstly, do the high-level properties, such as the narrative or event structure of the movie, increase or decrease attention as measured by secondary reaction time (RT) in a film? This was also examined by looking at the slope of RTs over elapsed time. In other words are people more engaged if a film has a narrative and do people become more or less engaged over time while watching a movie? Secondly, what are the relative roles of low-level features and high-level properties on attention while viewing film?

The first question was investigated by comparing dual-task secondary reaction times for unshuffled (i.e., the original) and shuffled versions of the films. Then by comparing the slopes of dual-task secondary reaction times over the duration of the film, in both unshuffled and shuffled movie versions. We used shuffling to disrupt the high-level properties of the film, a method pioneered by Hasson, Yang, Vallines, Heeger, & Rubin, 2008. One important part of the shuffling manipulation was that for each of the media segments used as the units for shuffling, the dual-task probe signals were positioned midway between the start and end of the segment. This meant that all visual properties of the film surrounding the probe position remained constant. This allowed the investigation of the roles of the higher-level factors independent of the low-level features. There were two distinct and opposing predictions. The first, being that an engaging narrative leads to higher cognitive load over time because increased engagement in a narrative leads to transport and attentional narrowing (Bezdek & Gerrig, 2016). The specific prediction here being that there would be steeper slopes of reaction times over elapsed time for the secondary task while watching an unshuffled film compared to the shuffled film. The opposing prediction being that poor narrative structure leads to steeper slopes of attention over elapsed time, because the event schemas are disrupted in a movie by shuffling, which will require more cognitive resources to process the movie (Brewer & Dupree, 1983; Gernsbacher, 1997; Zacks et al., 2007). The specific prediction here being that there will be steeper slopes of reaction times over elapsed time for the secondary task while watching the shuffled film compared to the unshuffled film.

The second question concerned the relative contribution of the low-level, audio-visual features on attentional engagement. This was investigated by processing the film using the iNVT (Itti et al., 2014) saliency model to obtain a set of time-dependent visual feature variables. Using these variables we carried out a comprehensive multiple regression analysis. This analysis was exploratory in nature and so did not seek to predict exactly which features determined behavior but rather asked the extent to which such features taken together could account for behavior in our task.

## Method

### Participants

Eighty participants took part in the experiment. All participants had normal vision and were studying experimental psychology at the University of Bristol, UK and completed the experiment for course credit. There were four groups of 20 participants. In the first group, *The Good Unshuffled group*, there were seven male participants whose ages ranged from 18 to 27 years (M = 22 years). In the second group, *The Good Shuffled group*, there were five male participants whose ages ranged from 18 to 27 years (M = 23 years). In the third group, the *About Time Unshuffled group*, there were eight male participants whose ages ranged from 18 to 32 years (M = 24 years). And in the fourth group, the *About Time Shuffled group*, there were five male participants whose ages ranged from 18 to 23 years (M = 20 years).

### Materials and design

The experiment was controlled by the SR Research Eyelink (Mississauga, ON, Canada) running Experiment Builder software 1.10.1385. The stimuli were displayed on a 17.3-in. Full High Definition LCD monitor (Dell Inc. Round Rock, TX, USA,), with a screen refresh of 50 Hz; spatial resolution of 1920 × 1080 pixels. Viewing distance was 75 cm (16 degs), for all participants.

In the study participants watched the first 40 min of one of two very different films: *The Good, The Bad and The Ugly* (Leone, 1967) – henceforth referred to as *The Good*, or *About Time* (Curtis, 2013) – henceforth referred to as *About Time*. These two films were chosen because they had wide appeal, and had similar mainstream Hollywood editing style structure in terms of editing conventions (Smith & Henderson, 2008). *The Good* is a fast-paced Western, with a strong narrative, and strong emotional engagement. The film was largely unknown to undergraduate students, so a pool of naïve participants could easily be recruited from the student population. In contrast, *About Time* is slower-paced, a romantic comedy, with a slightly confusing, narrative structure, as the main protagonist plays with time travel to try and win the girl. This film again is generally unknown to undergraduate participants. Previous work on film (Smith, 2012; Cutting et al., 2010; Zacks et al., 2007) has shown that editing structures might be responsible for modulating attention, so it is interesting to consider the shot structure in our chosen films. *The Good*, has an average shot length of 3.7 s with a standard deviation (SD) of 8.7 s, with a minimum shot length of 0.1 s (taken from the published values on (Cinemetrics, 2018), and a maximum shot length of 87.2 s (Cinemetrics, 2018). *About Time* has an average shot length of 50 s, a SD of 39 s, with a minimum shot length of 3 s and maximum shot length of 176 s (Cinemetrics, 2018). Within this study, attentional probes are used to make measurements at 15-s intervals, the irregular pattern of the cuts within these films should not, therefore, lead to any systematic biases.

There were four between-participant groups in the experiment. The first group, *The Good Unshuffled group,* watched the first 40 min of the film, *The Good* (Leone, 1967) in the original order. The second group, *The Good Shuffled group* watched the first 40 min of the same film in a shuffled order. The third group, the *About Time Unshuffled group,* watched the first 40 min of the film *About Time* (Curtis, 2013) in the original order. The fourth group, the *About Time Shuffled group,* watched the first 40 min of the same film in a shuffled order.

Concerning the sound track, one approach would be to remove the sound track from the film, and just play participants the visual component. However, at the very least this approach is likely to disrupt the narrative of the film; at the most extreme the film might no longer make sense. So for the current study we have chosen to include the sound track and play the film in the form in which it would be normally watched and enjoyed. For all the unshuffled films, audio-tones were mixed into the sound track of the film at regular 15-s intervals for all films. For each temporal position, the tone frequency was randomly chosen to be either a high tone (1000 Hz) or a low tone (600 Hz). The film stimuli were the same for all participants. The sound was played via a set of stereo headphones with the volume set to a comfortable listening level.

For the shuffled versions of the films, we started with each of the unshuffled versions of the film (video and sound including the added audio-tones), made working copies and then digitally cut the film into 15-s segments using a Matlab script, by bisecting mid-way in time between the regularly spaced audio-tones. The media segments were then randomly ordered, similar to perfectly shuffling a pack of cards, using the Knuth Algorithm P algorithm (Knuth, 1997), giving a shuffled version of the movie. As a result, within the 7.5 s either side of the onset of the audio-tone, the film imagery and sound were identical to the corresponding unshuffled film segment. With this manipulation the low-level features are kept constant, but the high-level narrative was disturbed.

### Procedure

Participants were told that their main task was to watch and enjoy the film, after which they would have to answer some questions to foster attentiveness to the movie (the questions were never asked). Participants were also told that while watching the film they should respond, on hearing either a high or low tone, by pressing either the “h” key or “l” key on the keyboard, respectively, and to do so as quickly but as accurately as possible. Participants were played sample audio-tones at the start, so that they could subsequently discriminate between high and low tones.

### Data analysis

The data analysis was carried out in two parts, addressing the two research questions.

The first data analysis was to investigate the effect of high-level factors on dual-task secondary RTs over elapsed time. The approach here was to investigate changes in RTs over time from each of the conditions by plotting RTs against elapsed time, and comparing the slopes. Comparison of the unshuffled and shuffled conditions for each film allowed the effects of narrative to be explored. Additionally, the RTs from the shuffled conditions were returned to their narrative position on the elapsed time axis – in a process that we have called *deshuffling*. By plotting deshuffled RTs against elapsed time, any trend in attention that related to the media properties, rather than the narrative order, could be examined. Finally, the deshuffled RTs were plotted against the shuffled RTs to allow common variance between these two to be examined; in other words do any trends in attention over time, in the shuffled films come from the lower-level features’ properties? Mean values of the individual secondary reaction times were calculated for each group and plotted against time and then compared with the predictions from a linear regression, performed using SPSS. It should be noted that the overall effect on RTs of shuffling the films will be examined through the comprehensive regression described in the second-stage data analysis.

The second data analysis investigated the relative contribution of the low-level, audio-visual features and high-level properties on the dual-task secondary reaction times. The films were computationally processed by the iNVT (Itti et al., 2014) software to derive 72 saliency-feature variables. Additionally, the audio sound track was processed by a Matlab script to derive a single audio-feature variable reflecting root mean square (RMS) loudness (Cutting, DeLong, & Brunick, 2018).The regression model was populated using the 72 saliency-feature variables, the RMS variable, the film choice (*The Good* or *About Time*), whether the film was shuffled or not and elapsed time. The SPSS Backwards elimination regression method was selected (see Field, 2009). More details are provided within the “Results” section.

## Results

Error rates across all conditions and participants were very low and so were not be analyzed further. For *The Good unshuffled group*, the mean error rate was 0.7%, range across participants 95% CI (0.658, 0.742); for *The Good shuffled group* the mean error rate was 0.6%, range 95% CI (0.557, 0.643); for the *About Time unshuffled group* the mean was 0.3%, range 95% CI (0.121, 0.421) and for the *About Time shuffled group* the mean was 0.4%, range 95% CI (0.161, 0.541).

Our initial analysis investigated the extent to which secondary RTs over the course of the film were consistent across participants. The justification for this was due diligence to test the construct validity of the secondary reaction-time measurements. We calculated the correlation in RT between every participant pair and reported the mean of these correlations in each condition. For *The Good unshuffled group* the mean inter-subject Pearson’s correlation was*,* $\overline{r}$ = .238, 95% CI (0.222, 0.254); for *The Good shuffled group*, $\overline{r}$ = .117, 95% CI (0.098, 0.136); for the *About Time unshuffled group*, $\overline{r}$ = .111, 95% CI (0.093, 0.129) and for the *About Time shuffled group*, $\overline{r}$ = .218, 95% CI (0.203, 0.234). There are reliable inter-participant correlations of secondary reaction times within each group.

**Fig. 2:**
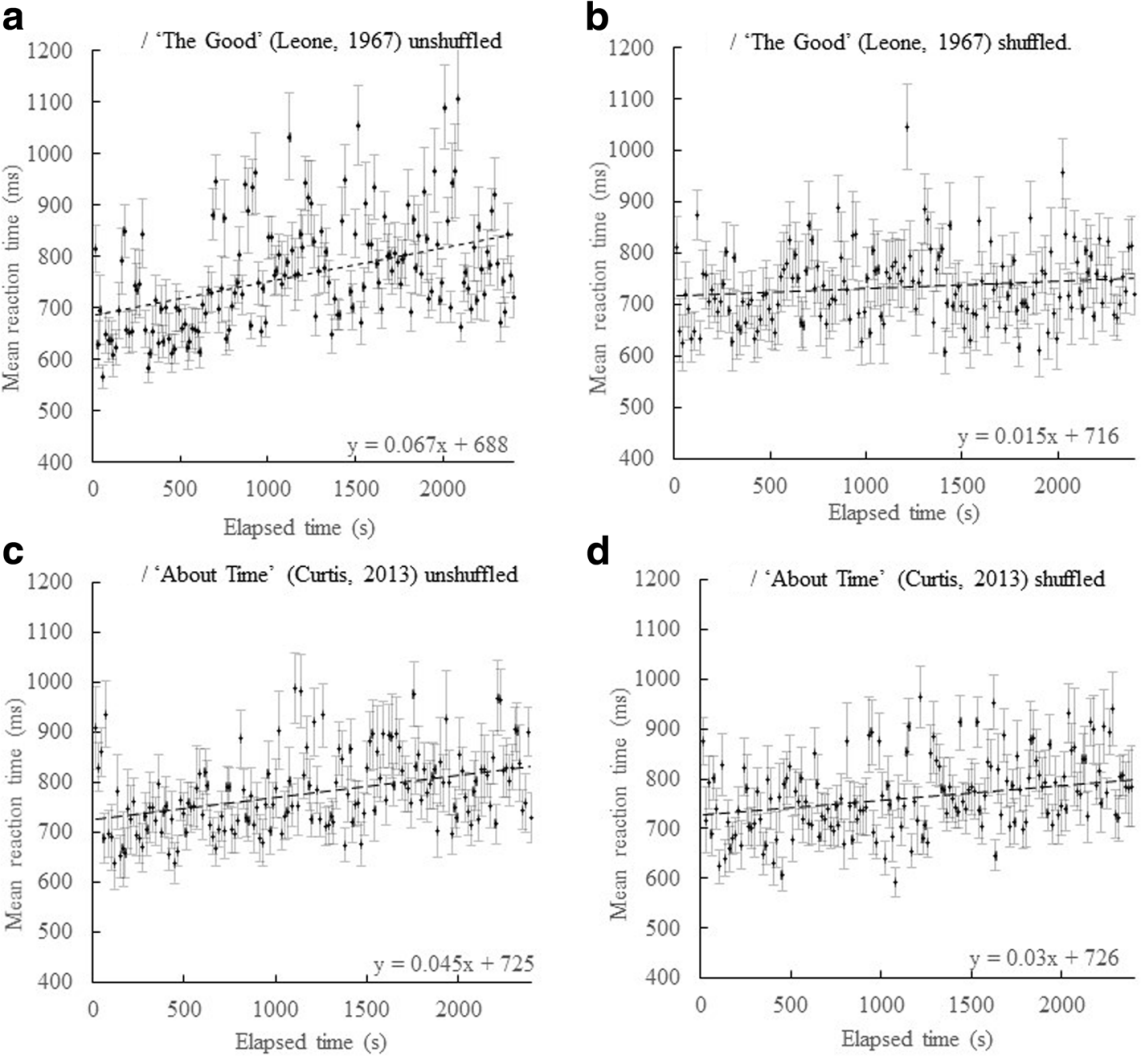
Mean secondary reaction time (RT) (ms) plotted against elapsed time (s), *N* = 20. **a**/ The Good unshuffled group. **b**/ The Good shuffled group. **c**/ About Time unshuffled group. **d**/ About Time shuffled group. Error bars are 95% CI

Next we proceeded to examine the slopes of the dual-task secondary reaction times for the four conditions. A graph of mean RT plotted against elapsed time for *The Good unshuffled group* is shown in Fig. 2A and for *The Good shuffled group* in Fig. 2B. A graph of mean RT plotted against elapsed time for the *About Time unshuffled group* is shown in Fig. 2C and the *About Time shuffled group* in Fig. 2D. Visual inspection of the unshuffled film conditions shows that the mean RT tends to increase with elapsed time but less so for the shuffled group. Linear regressions for each of the four conditions show slopes, (ms. over the 40-min duration of viewing) of 161 ms 95% CI (106, 216) for *The Good unshuffled group*; 36.0 ms, 95% CI (0, 74.4) for *The Good shuffled group*; 108 ms, 95% CI (69.6, 146) for the *About Time unshuffled group* and 72 ms, 95% CI (21.6, 122) for the *About Time shuffled group*. The slopes are larger for the unshuffled condition than the shuffled one. In the case of *The Good* shuffled condition, confidence intervals (CIs) suggest that the slope does not differ from zero. For *About Time*, CIs suggest that the slope is above zero, but still lower than the unshuffled value, i.e., there is a reduction in the slope value of 36 ms over the 40 min. of elapsed time. However, given the CI values, the *About Time* findings are neither significantly different, nor reduced to zero as is the case for the film *The Good*.

**Fig. 3:**
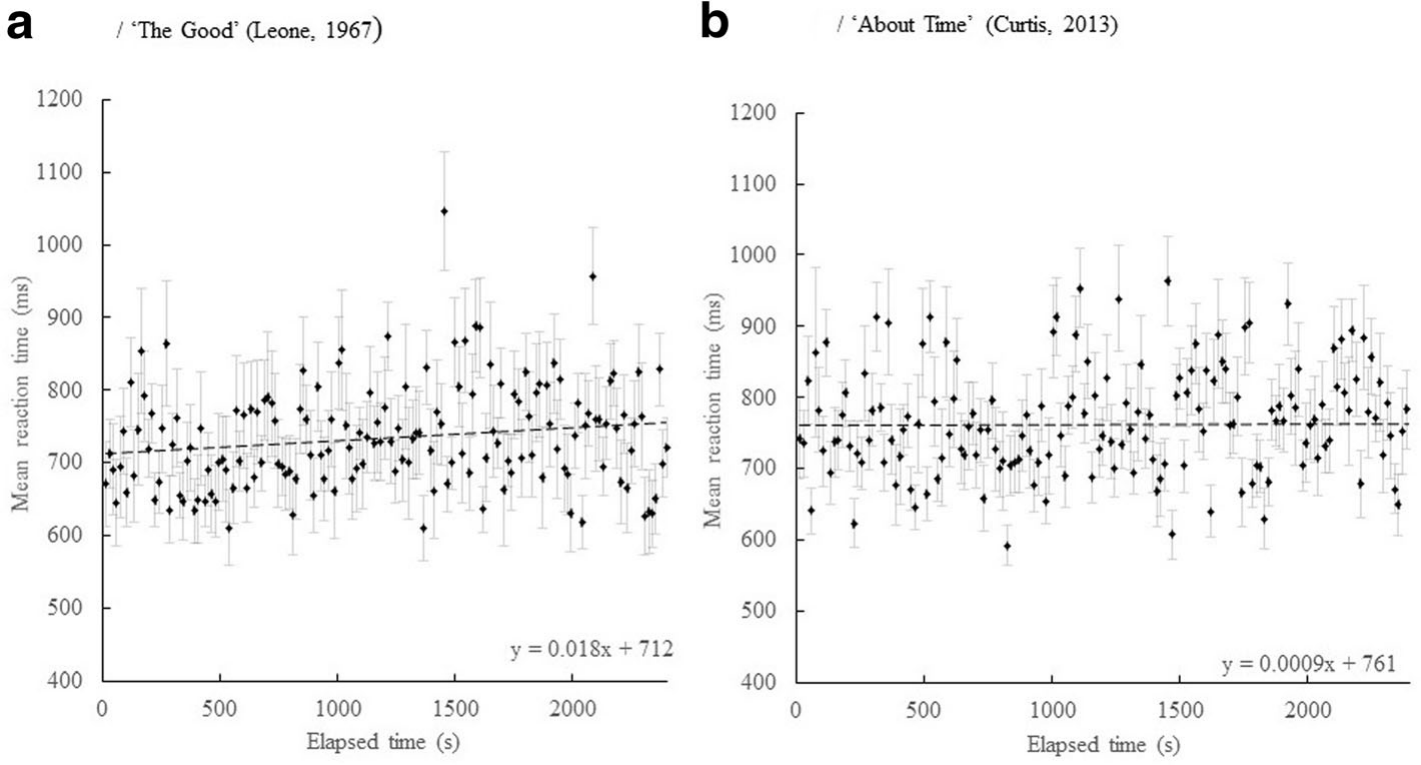
Deshuffled films, mean secondary reaction time (RT) (ms) across participants, for each time point, plotted against elapsed time (s). **a**/ The Good. **b**/ About Time. Error bars are 95% CI

In order to further explore the shuffled data, the reaction times were *deshuffled*, a process that reordered the reaction times to their original positions with respect to the elapsed time of the movie in the unshuffled films. These data are plotted in Fig. 3, which shows the deshuffled mean secondary RT plotted against elapsed time for A/ *The Good* (Leone, 1967); and B/ *About Time* (Curtis, 2013); Linear regressions for the two slopes (ms. over the 40-min duration of viewing) of 43.2 ms, 95% CI (7.2, 81.6) for *The Good* deshuffled and 2.4 ms, 95% CI (− 50.4, 55.2) for *About Time* deshuffled. It appears then that the increase in response time across viewing in the unshuffled condition is not solely a property of the local properties of the film.

**Fig. 4:**
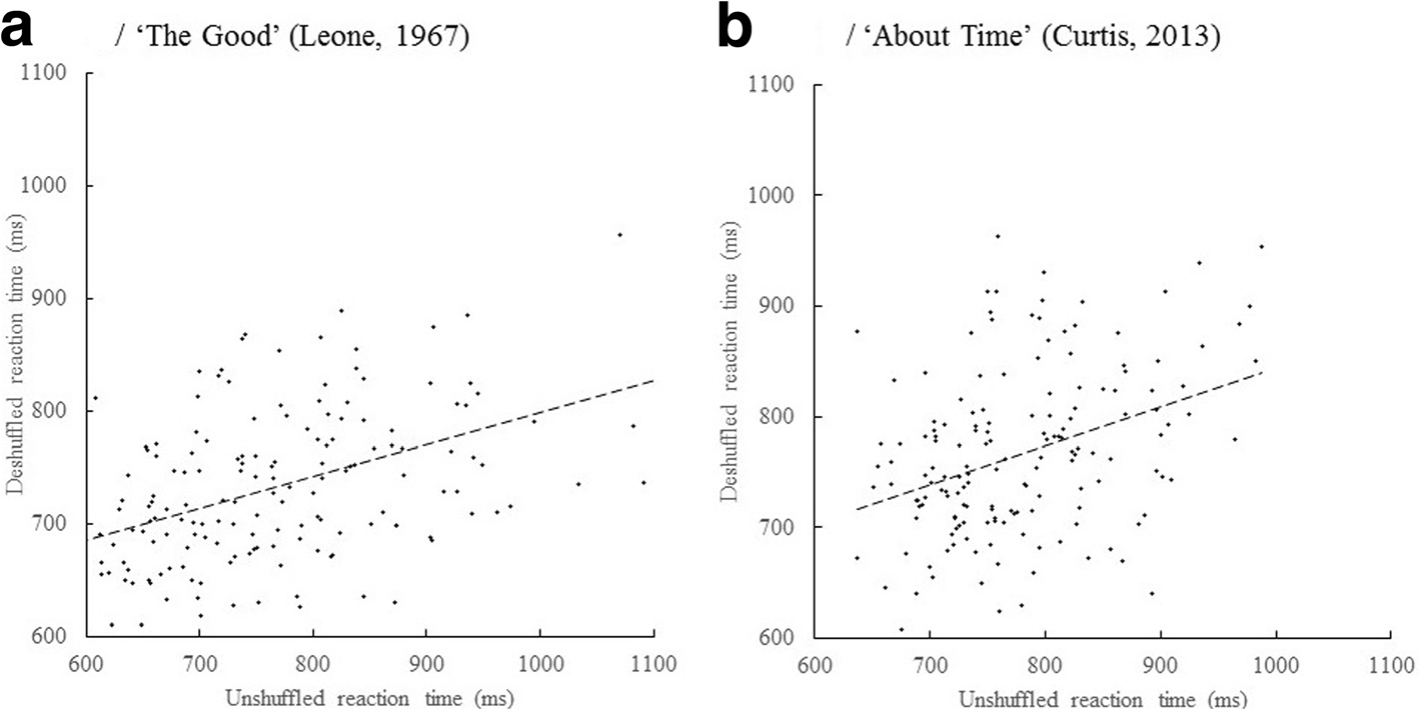
Deshuffled mean secondary reaction time (RT) (ms) plotted against the unshuffled mean secondary RT (ms). **a**/ The Good. **b**/ About Time. Error bars are 95% CI

We next plotted these deshuffled data against the unshuffled data values, as shown in Fig. 4A and B, in order to determine if there is a common component between RTs in the unshuffled and shuffled order. Performing a correlation between the mean RT deshuffled and unshuffled data for both films was highly significant, *p* < .001, for *The Good*, *r* = .456, and for *About Time*. *r* = .361. The probed mean RTs that are correlated are from the identical points in the source movie, but were presented at different elapsed times in the experiment in the shuffled compared to the unshuffled condition. Hence, any correlation has to be due to local media features and cannot be due to order/time effects. This shared component between the unshuffled and shuffled conditions supports the idea that some of the variability in response times in the unshuffled case is a result of the local context around the time of the presentation of the probe. Together these results suggest a role for both the broader narrative and the local context in determining response time.

### Modeling the role of low-level saliency

The analysis above suggests that a component of the variability in response times is a result of the local audio-visual context in which the probe was delivered, together with the narrative. To investigate this further, we carried out a regression analysis to predict reaction times using low-level perceptual features derived from the iNVT (Itti et al., 2014) biologically inspired model of vision.

As a first step, the film clips were processed, using the iNVT (Itti et al., 2014) saliency model with default settings, generating 72 saliency variables for: contrast, intensity, orientation, flicker and motion. The visual features were then further processed to produce 72 mean-feature variables, averaged over a 1-s window, prior to each of the dual-task probe points. The RMS loudness (see above) of the sound track for each film clip (and audio s/n of probe against the background) was also sampled in a 1-s window prior to the dual-task probe points, giving an additional feature variable for the regression.

A comprehensive linear regression (using backwards elimination, see Field, 2009) was then carried out using: the set of 72 visually derived variables, together with the single audio-feature variable (RMS), the film choice (*The Good* or *About Time*), together with a variable indicating whether the film was shuffled or not and the elapsed time.

The regression resolved after 51 iterations. The model explained 28% of the total variance with a Durbin-Watson coefficient of 1.70, which is considered indicative that collinearity was preserved (and that autocorrelation effects are not confounding the regression results). Analyzing the variance contribution using a block-wise regression, showed that the major predictors were low-level saliency factors accounting for 13% of the variance and time which accounted for 11% of the variance; the remainder was accounted for by audio RMS loudness, choice of film. It should also be noted that shuffling was a highly significant factor, with a negative slope showing that watching a film with an intact narrative structure leads to slower RTs than a shuffled film. In other words, significantly higher attentional engagement. Comparing the unshuffled and shuffled models, the narrative effect gave a difference of 8% in the variance. A summary conclusion is that increased saliency of the scene leads to slower RTs, i.e., increased processing.

Examining the comprehensive regression model, elapsed time was a highly significant coefficient, *t* = 9.95, *p* < .001, indicating that there was an increasing RT as the films progressed. Shuffling was a highly significant factor, *t* = − 4.65, *p* < .001 with a negative coefficient, indicating that shuffling the films, i.e., disturbing the narrative flow of the media, reduced the RT, in other words unshuffled films are more engaging than shuffled films. The choice of film itself was a highly significant factor, *t* = 3.14, *p* < .002, RTs were overall slower for *About Time* than *The Good*. This suggests that different films and hence different prolonged activities will lead to different attentional profiles. In terms of the audio, the loudness of the sound track (RMS), was a highly significant factor, *t* = 4.92, *p* < .001, the louder the film the longer the RT. This is indicative that audio is an engaging experience for the participant, but also might indicate that the loudness of the audio may mask the tone.

A number of individual visual features of the model are also significant coefficients (see the “Appendix” for a full breakdown); however, we would caution the reader from making too much of the exact pattern here for two reasons. First, there was no clear pattern as to which class of visual-feature-determined RT. Out of the 72 visual features included in the regression, significant features include: eight motion features, three intensity features, two blue-yellow color-opponency features, four orientation features and one flicker feature. Second, the spatial occurrence of these individual features is unlikely to be independent in the movies. However, we have included the breakdown in an Appendix as these patterns from the current exploratory study may provide an important starting point for future hypothesis-driven research.

## Discussion

The aim of this experiment was to investigate dynamic attention over prolonged time periods, by having participants engage in the continuous, compelling and naturalistic activity of watching movies. This contrasts with the many historic studies of attention that looked at short duration trials with repeated relatively simple and short stimuli. The dual-task secondary reaction-time paradigm was used to measure attentional capacity at periodic intervals, giving a dynamic measure of attention or engagement over time. This study focused on two main research questions: Firstly, to what extent is attention determined by a film’s high-level factors such as narrative and event structure? And secondly, what are the relative contributions of low-level, audio-visual features and high-level features on attentional engagement over time?

For the first of these questions, it is clear that there was an increase in the slope of attention over time, for a normally ordered film. Additionally, there was an increase in dual-task secondary reaction time for the unshuffled films compared to the shuffled films, which is consistent with the casual observation that audiences become more absorbed in the narrative of a film over time. These results, however, are in agreement with other studies that suggest that audiences become more immersed in film over time (e.g., Hasson et al., 2004; Mital, Smith, Hill, & Henderson, 2011, and Smith, 2012). As expected, shuffling the films reduced this trend, consistent with the idea that this trend is not a result of fatigue over time but rather changes in engagement in the higher-level properties of the movie. Note, however, that comparing the deshuffled to unshuffled film stimuli showed that a component of shared variance existed, suggesting that low-level features regardless of narrative also drive attention. This result confirms the predictions that increased engagement in the narrative leads to transport and attentional engagement (e.g., Bezdek & Gerrig, 2016) and is contrary to the opposing hypothesis that an incoherent or broken narrative will lead to a great attentional load in order to construct sense in the content via event processing (Brewer & Dupree, 1983; Gernsbacher, 1997; Zacks et al., 2007).

The second research question concerned the relative contributions of the low-level features and high-level properties on attentional engagement. The regression model including all the features for all four conditions was able to predict a large proportion of the variance (roughly a quarter) in the dual-task reaction time. As outlined above we do not particularly want to focus in on which features drove these effects but only to note here that a wide mix of low-level features were found as predictors of attention from the saliency model. Elapsed time was also a major factor in the regression, confirming the findings from the film-by-film analysis. Other regression factors that contributed to the variance included, shuffle, film choice, and loudness of the audio sound track. Film choice was a reliable factor on the regression model. It should be noted that we chose two films from very different genres here and this result indicated that there is an interesting stream of future work that could be carried out investigating the effect of different movies on attention. Such research would require the testing of a much wider range of movies and a clearer understanding of how to classify these films a priori. But clearly, the current methods have the sensitivity to detect such differences.

The loudness of the audio sound track was also a reliable factor in the regression. The modeling conducted in this study focused largely on the visual features of the film and neglected to comprehensively model the audio features. This is clearly a topic for future research but was beyond the scope of the current study. One comprehensive list of audio features is described in recent thesis work by Mital (2014) on decomposing and reforming soundscapes. Mital (2014) uses a sound feature set including Mel-Frequency Cepstral Coefficients (MFCCs) and low-level psychoacoustic descriptors’ spectrum power, centroid, zero-crossing rate, brightness and pitch. MFCCs were originally applied to speech recognition (Davis & Mermelstein, 1980).

In these analyses we have chosen to emphasize overall variance due to low-level features. The pattern that emerged from the regression about the specific features that drive attention is complex. However, future research could build on this regression approach, and build different regression models, which are theory driven and more open to a more meaningful quantitative analysis of features. It should also be noted that the features that predict reaction time may vary across films – this is clearly a rich topic for future work for which the current paper lays the foundation.

It is interesting to draw parallels between the current study and the study by Loschky, Larson, Magliano, and Smith (2015). Both manipulate film to create two conditions one with a strong narrative and one with a weakened narrative. The Loschky et al. study uses eye movement synchronization similarity rate to gauge audience synchronization. Whereas the current study compares a dual-task measurement of attentional load. The Loschky et al. study found that attentional synchronization is high in both conditions, but slightly stronger when narrative is present. This suggests that with a strong narrative more people will be paying attention to the same place at the same time, which could be taken as high immersion. This agrees with the findings from the current study, given that higher attention load indicates higher engagement or immersion. This is also reminiscent of an early neuro-cinematics study by Hasson et al. (2004) presenting neurophysiological evidence for synchronization of viewer attention across an audience as inter-subject correlation measured through the functional magnetic resonance imaging (fMRI) BOLD signals.

## Conclusions

This study showed that both low-level features and high-level narrative factors drive dynamic attention to a continuous prolonged stimulus with naturalistic properties, in this case film. The dual-task secondary reaction-time paradigm proved a valid way of measuring such fluctuations in attentional engagement over elapsed time, and would be a useful general method in future studies of attention to prolonged stimuli. We would suggest that future researchers might consider applying this method to scenarios such as natural viewing conditions in the real world, other types of film media, Virtual Reality and Augmented Reality. Also given these results, it is perhaps timely to review early pioneering psychological studies, which were necessarily faced with the limitations of early equipment, and experimental methods, and, therefore, had to simplify conditions in the laboratory and use repeated, short, simple stimuli. This study, we believe, garners support to considering attention in terms of a stream of experience, rather than discrete moments. And that prolonged patterns of attention may be hidden by using short experimental tasks and can only be captured by using a continuous naturalistic stimuli such as film.

## Appendix

### Regression results

The table below gives the significant unique feature variables resulting from the backwards regression, with their standardized Beta, and *p* values. Multiple visual-feature variables are shown for each type as derived from the iNVT (Itti et al., 2014) model. Detectors are described by the feature they respond to, their preferred orientation (0°,45°, 90° and 135°), and the spatial scale over which they are sensitive (coarse, medium and fine). The full SPSS regression output is included in supplementary data, with the full, and somewhat technical Itti and Koch (1998) feature notation.

**Table 1:** SPSS regression table giving Beta coefficients and *p* values

Feature	Beta	*p value*
Time	.351	.000
RMS	.188	.000
Shuffle	−.160	.000
Film	.190	.002
Motion, medium, 45°	.340	.002
Orientation, coarse, 90°	.131	.003
Motion, coarse, 45°	.252	.004
Intensity, coarse	1.92	.004
Intensity, medium	−1.77	.007
Motion, medium, 90°	−.356	.007
Blue-yellow, coarse	.242	.009
Orientation, medium, 45°	.159	.010
Motion, coarse, 45°	−.259	.011
Motion, coarse, 45°	−.311	.012
Orientation, coarse, 45°	−.128	.017
Blue-yellow, medium	−.217	.019
Orientation, coarse, 45°	−.119	.021
Intensity, coarse, fine	−.214	.027
Motion, coarse, 45°	.277	.022
Motion, coarse, 45°	−.166	.031
Motion, coarse, 90°	.239	.032
Flicker, medium	−.105	.042

*RMS* root mean square
